# A Simple Bayesian Method for Evaluating Whether Data From Patients With Rheumatic Diseases Who Have Been Under Chronic Hydroxychloroquine Medication Since Before the COVID-19 Outbreak Can Speak to Hydroxychloroquine's Prophylactic Effect Against Infection With SARS-CoV-2

**DOI:** 10.3389/fmed.2020.00490

**Published:** 2020-08-13

**Authors:** Serban C. Musca

**Affiliations:** Université Rennes 2, Campus Villejean, Place du Recteur Henri Le Moal, CS 24307, Rennes Cedex, France

**Keywords:** SARS-CoV-2 / COVID-19, hydroxychloroquine, chronic medication, prophylactic effect, systemic lupus erythematosus, rheumatoid arthritis, clemastine, cloperastine

## Abstract

No vaccine against infection by SARS-CoV-2 yet exists. Treatment by hydroxychloroquine (HCQ) medication, among others, has been proposed. However, prophylactic HCQ medication has been little evaluated. We propose to use data from patients with rheumatic diseases (RA, SLR) who have been chronically taking HCQ medication since before the COVID-19 outbreak (hereafter: HCQpa), in order to evaluate the potential of HCQ for preventing infection with SARS-CoV-2. This can be achieved with relative ease by considering whether COVID-19 prevalence is significantly lower in HCQpa than in the general population (i.e., all people that are not HCQpa). Even if COVID-19 prevalence is truly significantly lower in HCQpa, some HCQpa may still present with COVID-19 (lower prevalence does not mean a prevalence of zero). However, given a value for COVID-19 prevalence in the general population and a number of available HCQpa, one may compute the maximum number of HCQpa for that total number of HCQpa considered that can have COVID-19 in order to still be able to conclude a lower COVID-19 prevalence in HCQpa (i.e., if there is one more case of COVID-19 than that maximum number, the COVID-19 prevalence in the HCQpa cannot be said to be lower than in the general population). Because the COVID-19 prevalence in the general population is not known with precision, we will consider different general population prevalence values. Among these contemplated prevalence values, one is derived from the official total number of confirmed cases, others by computing the total number of cases from the number of fatal COVID-19 cases so far and considering different case fatality rates per total cases. Our analyses show that systematic testing for COVID-19 in as few as 5,000 HCQpa is all that is needed for a test of whether HCQ has a prophylactic action against COVID-19, even for a COVID-19 prevalence value as low as 250 per 100,000, provided that test sensitivity is at least equal to its specificity. For higher COVID-19 prevalence values, the number of HCQpa needed is even lower.

## Introduction

We all know that “we do not have antivirals, vaccines, antibody-based therapeutics, or specific treatments” (1) with which to avoid infection with severe acute respiratory syndrome coronavirus 2 (SARS-CoV-2) and to treat against coronavirus disease 2019 (COVID-19). Hydroxychloroquine (HCQ) medication has some effectiveness against COVID-19 *in vivo* (2–4) during the early symptomatic phase (3) and also in the long run, possibly because it “contribute[s] to attenuating the inflammatory response.” (5) HCQ is not a new medicine, and clearly defined drug safety management recommendations for it exist (6). Given the global situation and the ongoing debate over whether HCQ medication is effective as a prophylactic means against SARS-CoV-2 and/or as a cure for COVID-19, we deemed worth exploring the feasibility of testing such a hypothesis. The questions that we ask here are as follows. Is there a large enough number of identifiable people who have been chronically taking HCQ medication since before the outbreak of SARS-CoV-2 as a treatment for other diseases? Is the number of those people large enough to allow for sound statistical inference? What results found in them would be suggestive of a prophylactic effect of HCQ against COVID-19?

A considerable number of people have indeed been chronically taking HCQ medication as a treatment for other diseases since before the outbreak of SARS-CoV-2. Thus, one could derive crucial information on the prophylactic effect of HCQ against infection with SARS-CoV-2 by analyzing data from patients chronically treated with HCQ since before the COVID-19 outbreak (hereafter: HCQpa). Indeed, HCQ is the treatment of choice for systemic lupus erythematosus (7) (SLE) and is also used as a drug in the management of rheumatoid arthritis (8) (RA). SLE prevalence is variable but is as high as 0.241% in the USA (9). RA prevalence is 0.24% globally (10) but is 0.5–1% in Europe and the USA (11, 12). The total number of HCQpa in a country with a population of millions thus constitutes a large, statistically interesting sample.

It may, of course, be that SLE, and RA patients are intrinsically more prone to infection in general and, in particular, with SARS-CoV-2, than all comers. However, finding that HCQpa are *less* prone to infection with SARS-CoV-2 than all comers who do not take HCQ medication would tend to prove that HCQ helps avoid infection with SARS-CoV-2.

## Methods

If HCQ has no prophylactic effect against infection with SARS-CoV-2, COVID-19 prevalence in HCQpa will not be statistically different from that in the general population (all comers who do not take HCQ medication; hereafter: pop_gen_).

Inferential statistics allow an informed decision to be made based on data and allow a statement (e.g., “medicine X is effective against disease Y”) to be made with a given degree of confidence. That degree of confidence is expressed as a probability and is usually 0.95. This probability of 0.95 means that when drawing a conclusion based on data, one has 95% chances of being correct and 5% chances of an erroneous (although suggested by the data) conclusion. If one wants to lower the chances of an erroneous conclusion, one can opt for a higher probability, for instance, 0.99. This is the value we opted for here.

If one wants to speak of the exact COVID-19 prevalence in HCQpa, one would have to test all HCQpa for SARS-CoV-2 infection in order to come up with an exact prevalence figure. Alternatively, one will have to test only a given number of HCQpa and express the result not as a value, but of an interval of values, because of the probabilistic nature of statistical inference. We decided to contemplate the more practicable second option and adopted the Bayesian credibility interval (13) as the interval of values used to draw our conclusions. The 0.99 credibility interval is an interval such that there is a 99% chance that the true value of the parameter under examination (here, COVID-19 prevalence in HCQpa) falls within its upper and lower bounds.

HCQ having a prophylactic effect against SARS-CoV-2 infection would manifest itself by a COVID-19 prevalence in HCQpa that is *lower* than the COVID-19 prevalence in the general population. Accordingly, we are interested in an *upper* bound of the COVID-19 prevalence in HCQpa 0.99 credibility interval that has a value that is still significantly *lower* than the COVID-19 prevalence in the general population.

As prevalence is “the proportion of cases of a specified condition that are fatal within a specified time” (14), that is

$$\begin{array}{l} Prevalence=\frac{TNC}{PS} \end{array}$$

with TNC being the total number of COVID-19 cases so far and PS the population size, one can reason in terms of TNC instead of prevalence (since PS is a constant). That is, for any given number of HCQpa, one can search for the maximum HCQpa TNC number that gives HCQpa COVID-19 prevalence a value that is still under the lower bound of a 0.99 credibility interval build based on the pop_gen_ COVID-19 prevalence value. If that TNC number is not reached (i.e., there are fewer COVID-19 cases for the total number of HCQpa considered), then one can conclude with a <1% chance of error that HCQ has a prophylactic effect against infection with SARS-CoV-2. The upper bound of the HCQpa TNC 0.99 credibility interval built on the pop_gen_ COVID-19 prevalence for a number N of HCQpa is given by finding the maximum value of TNC in HCQpa such that

$$\begin{array}{l} 1-pbeta(\text{po}{\text{p}}_{\text{gen}}\text{COVID-}19\text{ prevalence value},\text{ TNC in}\\ \text{ HCQpa},\text{ N}-\text{TNC in HCQpa})<0.005 \end{array}$$

with *pbeta* being the cumulative probability function of a beta distribution (15, 16).

In the absence of systematic detection of COVID-19 in all of the individuals in a population, TNC is underestimated. Thus, computing the prevalence based on the reported TNC will result in a (vastly) underestimated prevalence value. To take that into account, we considered different TNC values. Because TNC itself is not meaningful, we considered different fatality rates per total cases, “the proportion of cases of a specified condition that are fatal within a specified time” (14) (hereafter: CFRTC), as the number of fatal COVID-19 cases so far (hereafter: NFCT) is known (14), and we know that

$$\begin{array}{l} TNC\;=\frac{NFCT}{CFRTC} \end{array}$$

## Results

In order to make this data simulation more concrete and personalized, we chose as an example country France, a European country that is affected by COVID-19 and has a population of around 65 million. As to the HCQpa that one would have to test if a study such as this one were actually run, it should ideally come from stratified sampling among the HCQpa. The data simulation results are based on the following data: NFCT = 22,890 and TNC_reported_ = 161,665 (17); PS_France_ = 65,241,000 (18). Considering a conservative sum of SLE and RA prevalence of 0.6% (the exact figure may be higher, perhaps more than double that which we consider here, but we only intend to find out whether the prevalence sum translated in terms of the total number of cases yields a number large enough for inference purposes, so we consider the more conservative figure of 0.6%) yields about 400,000 SLE and RA patients in France. Supposing one in five of them has been medicated with HCQ entails a number of HCQpa of about 80,000. We also consider other, lower values for the number of HCQpa in order to test for the limits of the method.

Tables 1, 2 show the results for the case where the SARS-CoV-2 detection test has the same sensitivity and specificity value. The more general case (i.e., different sensitivity and specificity values) is discussed afterward.

**Table 1:** Maximum HCQpa TNC number such that one can conclude with <1% chance of error that HCQ has a prophylactic effect against infection with SARS-CoV-2 (MaxTNC_.99_), as a function of case fatality rate per total cases (CFRTC) and the number of available HCQpa.

**CFRTC (%)**	**TNC**	**COVID-19 prevalence in the general population (per 100,000)**		**Number of available HCQpa**					
				**80,000**	**50,000**	**20,000**	**10,000**	**5,000**	**2,000**
14.159	161,665	247.797	**MaxTNC**_**.99**_	163	96	32	13	4	–
7	327,000	501.219		350	211	75	33	13	3
5	457,800	701.706		501	304	111	50	21	5
2.5	916,600	1,403.412		1,038	635	239	111	50	15
1.75426	1,304,824	2,000.006		1,499	920	350	165	75	25
1.16951	1,957,230	3,000.000		2,277	1,403	539	257	120	41
0.7017	3,262,078	5,000.043		3,842	2,375	921	445	211	76
0.58475	3,914,493	6,000.051		4,628	2,864	1,114	540	258	93

*See text for details*.

**Table 2:** Modal value for COVID-19 prevalence per 100,000 in HCQpa for the critical values MaxTNC.99 given in Table 1, as a function of case fatality rate per total cases (CFRTC) and the number of available HCQpa.

**CFRTC (%)**	**TNC**	**COVID-19 prevalence in the general population (per 100,000)**		**Number of available HCQpa**					
				**80,000**	**50,000**	**20,000**	**10,000**	**5,000**	**2,000**
14.159	161,665	247.797	**HCQpa modal COVID-19 prevalence**	202.505	190.008	155.016	120.04	60.024	–
7	327,000	501.219		436.261	420.017	370.037	320.064	240.096	100.100
5	457,800	701.706		625.016	606.024	550.055	490.098	400.160	200.200
2.5	916,600	1,403.412		1,296.282	1,268.051	1,190.119	1,100.022	980.392	700.701
1.75426	1,304,824	2,000.006		1,872.547	1,838.074	1,745.175	1,640.328	1,480.592	1,201.201
1.16951	1,957,230	3,000.000		2,845.071	2,804.112	2,690.269	2,560.512	2,380.952	2,002.002
0.7017	3,262,078	5,000.043		4,801.370	4,748.190	4,600.460	4,440.888	4,201.681	3,753.754
0.58475	3,914,493	6,000.051		5,783.895	5,726.229	5,565.557	5,391.078	5,142.057	4,604.605

*See text for details*.

With TNC_reported_ = 161,665, pop_gen_ prevalence is about 248 per 100,000 (see first line of Table 1). For a number of HCQpa of 80,000, finding up to (and including) 163 HCQpa with COVID-19 disease would lead one to rightfully conclude that COVID-19 prevalence in HCQpa is lower than in pop_gen_. Actually, the modal (the mean has no specific meaning attached to it and is thus uninterpretable for a beta distribution) COVID-19 prevalence in HCQpa in that case is of 202.205 per 100,000, as one can read in the corresponding cell in Table 2.

At the same pop_gen_ prevalence (i.e., same row in Table 1; here, first row, i.e., pop_gen_ prevalence of about 248 per 100,000), as the number of available HCQpa decreases from 80,000 to 50,000, to 20,000, etc., the maximum number of HCQpa that can be found with COVID-19 disease in order to still have a COVID-19 prevalence lower in the HCQpa than in pop_gen_ decreases, unsurprisingly. With only 2,000 HCQpa available, there is no way to evidence such a result, since even finding no HCQpa with COVID-19 disease does not warrant the inference that there is a lower COVID-19 prevalence in HCQpa than in pop_gen_. Inspection of Table 2 shows how the modal COVID-19 prevalence—computed for the maximum number of HCQpa that can be found with COVID-19 disease in order to still have a COVID-19 prevalence lower in the HCQpa than in pop_gen_—decreases as the number of available HCQpa decreases.

We will now consider different pop_gen_ prevalence values, that is, look always in the column of a given number of available HCQpa and consider how results change as one looks at different rows (different prevalence values). The reason to consider different pop_gen_ prevalence values is that pop_gen_ prevalence computation depends on TMC, and TMC is likely greatly underestimated in the absence of systematic testing because it is the ratio of NFCT (likely accurate) to CFRTC (likely greatly overestimated). Hypothesizing a different, lower CFRTC (while holding NFCT constant at 22,890) has the consequence of *increasing* pop_gen_ COVID-19 prevalence. For instance, supposing CFRTC = 7% puts TNC value at 327,000 cases. In turn, this affects pop_gen_ COVID-19 prevalence, now at about 501 cases per 100,000. We also consider other lower CFRTC values that correspond to reported values for other countries (i.e., 5 and 2.5%), which yield pop_gen_ COVID-19 prevalence values of about 700 and 1,400 per 100,000, respectively. A Belgian study (19) using stratified sampling, published on March 26, 2020, found that 3–6% of adult people had antibodies against COVID-19, so we also considered lower CFRTC values that yield higher pop_gen_ COVID-19 prevalence values in the range between 2,000 and 6,000 per 100,000.

If we consider the case where 80,000 HCQpa are available, one can see from inspection of Table 1 how the maximum number of HCQpa that can be found with COVID-19 disease in order to still have a COVID-19 prevalence lower in the HCQpa than in pop_gen_ increases dramatically with COVID-19 prevalence in pop_gen_. For instance, if COVID-19 prevalence in pop_gen_ were indeed 6,000 per 100,000, one can have more than one HCQpa in 20 (4,628 out of 80,000) presenting with COVID-19 and still correctly conclude that COVID-19 prevalence is lower in HCQpa than in pop_gen_—as one can see in Table 2, the modal COVID-19 prevalence in HCQpa would be then of about 5,784 per 100,000.

If we consider instead the case where only 2,000 HCQpa are available, the same is observed. Interestingly, however, one can see that for a COVID-19 prevalence in pop_gen_ as low as 500 per 100,000, it is possible now to evidence a COVID-19 prevalence that is lower than in pop_gen_. Moreover, at the other extreme of pop_gen_ COVID-19 prevalence values considered, 6,000 per 100,000, one can have more than one HCQpa in 23 (93 out of 2,000) presenting with COVID-19 and still correctly conclude that COVID-19 prevalence is lower in the HCQpa than in pop_gen_—but now, as one can see in Table 2, the modal COVID-19 prevalence in HCQpa would be about 4,605 per 100,000.

Now let us consider what changes when the SARS-CoV-2 detection test has sensitivity and specificity values that differ one from another. This has to do with the false-positive rate (FPR) and false-negative rate (FNR), respectively, of the test. Indeed, the FPR of a test is the probability that the test gives a positive result in the absence of what it is used to detect. If we denote T a positive test result and notS the absence of SARS-CoV-2, the FPR of the test is P(T|notS). On the other hand, the specificity of a SARS-CoV-2 test is the probability that it gives a negative result (notT) in the absence of SARS-CoV-2, P(notT|notS). One can now see that the FPR and specificity sum up to one, P(T|notS) + P(notT|notS) = 1, so the further away the specificity of a test is from one, the higher its FPR, that is, the higher the probability of erroneously labeling a healthy person as infected with SARS-CoV-2. Similarly, the FNR of a test is the probability that the test will give a negative result when the person is in fact infected with SARS-CoV-2, P(notT|S). The sensitivity of that test, its probability of detecting SARS-CoV-2 when the person is infected with SARS-CoV-2, is P(T|S). One can now see that the FNR and sensitivity sum up to one, P(notT|S) + P(T|S) = 1, so the further away the sensitivity of a test is from one, the higher its FNR, that is, the higher the probability of erroneously labeling a person infected with SARS-CoV-2 as healthy.

What happens if FNR and FPR have the exact same (non-zero) value? Some healthy persons will be erroneously labeled as infected with SARS-CoV-2 because FPR is different from zero, and some of the persons infected with SARS-CoV-2 will be erroneously labeled as healthy because the FPR is different from zero, but over a large number of persons being tested, the number of persons with SARS-CoV-2 the test has missed and the number of healthy persons the test has labeled as infected with SARS-CoV-2 will be the same so that the total number of persons labeled as infected with SARS-CoV-2 will be correct. As *FNR* = *1- sensitivity* and *FPR* = *1- specificity*, it is clear that *FNR* = *FPR* when *sensitivity* = *specificity*.

In order to avoid the pitfall of concluding a prophylactic effect of HCQ, it is important that the FNR be at most equal to the FPR. Indeed, if the FNR were higher than the FPR, one would miss more persons infected with SARS-CoV-2 than the number of healthy persons that one would erroneously include as persons infected with SARS-CoV-2. One thus wants to have FNR ≤ FPR, that is, *1- sensitivity* ≤ *1- specificity*, which in turn requires *sensitivity* ≥ *specificity*.

If *sensitivity* = *specificity*, one can use Table 1 directly. Otherwise, a correction has to be made to the values of MaxTNC_.99_ given in Table 1 (and the COVID-19 prevalence in HCQpa from Table 2 should not be used; instead, the correct COVID-19 prevalence in HCQpa would have to be recalculated using the corrected MaxTNC_.99_ value). If *specificity* ≥ (≤) *sensitivity*, then the correction entails subtracting (adding) *Number of available HCQpa* ^*^(specificity—sensitivity). For instance, for *sensitivity* = 0.995 and *specificity* = 0.9975, with 2,000 HCQpa and a COVID-19 prevalence of 1,403 per 100,000, the correction would entail subtracting 2,000 ^*^ (0.9975–0.995) = 5, so that MaxTNC_.99_ would now have to be 10 (instead of 15; see Table 1) — also, the COVID-19 prevalence in HCQpa would then be 450.450 (instead of 700.701 from Table 2) per 100,000.

## Discussion

This study is concerned with the feasibility of drawing conclusions as to the prophylactic effect of HCQ against SARS-CoV-2 by taking into consideration data from people suffering from a disease that forced them to chronically take HCQ medication since before the outbreak of SARS-CoV-2. Firstly, we have established that there is a considerable number of people who are in this situation, as HCQ is used chronically in the treatment of SLE and RA. Indeed, for a country such as France (i.e., with a population of about 65 million), supposing a conservative prevalence sum for SLE and RA of 0.6% yields about 400,000 SLE and RA patients, so it is reasonable to suppose that some tens of thousands among them have been chronically taking HCQ medication since before the outbreak of SARS-CoV-2 (we will refer to those people as HCQpa, and we estimated their number to be of about 80,000).

We focused most of this study on the question of what results one should find in HCQpa in order to infer a prophylactic effect of HCQ against SARS-CoV-2. The obvious answer is that to begin with, one must find a COVID-19 prevalence in HCQpa lower than in the general population (defined as the population of all people who do not take HCQ medication). We then considered a Bayesian inference method that gives the maximum number of HCQpa—among a given number of available HCQpa—that could have the COVID-19 disease such that COVID-19 prevalence in HCQpa is still found to be significantly lower than in the general population. Because there is no consensus as to the value of COVID-19 prevalence in the general population, we considered different such values.

The interesting question that then arises is whether the number of available HCQpa is large enough to allow a lower COVID-19 prevalence in HCQpa than in the general population to be evidenced for all, for some, or for none of the values of COVID-19 prevalence in the general population. Another important question, for obvious practical reasons, is the minimum number of available HCQpa necessary in order to be able to reach such a conclusion if it were true. Our results (see Tables 1, 2) show that the answer to the first question is affirmative and that the answer to the second is that quite few available HCQpa are needed.

This is a somewhat unsurprising result but is still interesting for its implication that, if COVID-19 prevalence is truly lower in HCQpa than in the general population, the higher the COVID-19 prevalence in the general population, the easier it is to evidence a lower COVID-19 prevalence in HCQpa than in the general population. With a SARS-CoV-2 detection test that has a sensitivity equal to its specificity, as few as 5,000 HCQpa would be enough to evidence a lower COVID-19 prevalence in HCQpa than in the general population (if that were true), even for a COVID-19 prevalence in the general population as low as 250 per 100,000. For a COVID-19 prevalence in the general population of 500 per 100,000 or higher, even a total of 2,000 HCQpa would be sufficient. Table 1 gives, for each considered COVID-19 prevalence value in the general population and for each number of available HCQpa, a critical value, that is, the maximum number of HCQpa who can present with COVID-19 such that COVID-19 prevalence will still be lower in HCQpa than in the general population; Table 2 displays the modal COVID-19 prevalence in HCQpa for those critical values.

We have also considered the case where the SARS-CoV-2 detection test has a sensitivity different from its specificity and, after analyzing the impact of this, we proposed a correction that, once applied, reduces this situation to that where the sensitivity of the test equals its specificity.

Finally, we consider the limits and potential extensions of the present study. A first limit, suggested by a reviewer, is that if the methodology we advocate here is put into practice and one does indeed find a lower COVID-19 prevalence in HCQpa than in the general population, there is no way to substantiate that the intake of HCQ by the HCQpa is the reason for the low prevalence of COVID-19. However, it seems to us difficult to argue that it is rather RA or SLR that instead protect against infection by SARS-CoV-2 (to our best knowledge, this has not been put forward in the literature). Still, the method outlined here applies to an observational type of study, and as such, even if the results show a lower COVID-19 prevalence in HCQpa than in the general population, one cannot conclude prophylactic effectiveness. To warrant such a conclusion, such findings ought to be confirmed in randomized clinical trials. To our best knowledge, however, the one published clinical trial (20) that aimed at testing for prophylactic effectiveness of HCQ and the others in progress or under review only tested *post-*exposure prophylaxis, that is, intake of HCQ following (i.e., subsequent to) exposure to SARS-CoV-2. The method we propose is to be used as a means to look for *pre*exposure prophylaxis; that is, it considers retrospective data inclusion in an observational study based on data from people who have been chronically taking HCQ medication since *before* the COVID-19 outbreak.

The methodology delineated here may be applied to other potential prophylactics or medication to test for their action against SARS-CoV-2. In that sense, the significance of the results presented here goes far beyond the question of whether HCQ may have a prophylactic effect and allows those who have been medicated with HCQ since before the COVID-19 outbreak to avoid infection with SARS-CoV-2. The only requirement is the existence of a large enough number of people who have been medicated with such potential drugs. Recently, after analyzing SARS-CoV-2 proteins and identifying which proteins from the human body they could interact with, a study (21) identified many drugs already approved for use in humans that could target those interactions and thus help avoid infection with SARS-CoV-2 or fight COVID-19. On that list, there are quite mundane drugs, such as the antihistamines clemastine, and cloperastine, which have antiviral activity against SARS-CoV-2, and that many people with allergies take chronically. A first test of the action of these drugs against SARS-CoV-2 could be done much more rapidly with the methodology that we presented here than in a clinical trial—the R script that was used to derive the results is made available in the Appendix, which is to be found on the online article page. Also, the evaluation of the feasibility of such a test has already been done here. Indeed, everything that was concluded here as to the feasibility of evaluating HCQ as a prophylactic against SARS-CoV-2 can be said *mutatis mutandis* for another drug that a large enough number of people take chronically.

## Data Availability Statement

Publicly available datasets were analyzed in this study. This data can be found here: John Hopkins Coronavirus Resource Center, https://coronavirus.jhu.edu/map.html, accessed April 26, 2020.

## Author Contributions

SM had the idea, did the analyses, and wrote the paper.

## Conflict of Interest

The author declares that the research was conducted in the absence of any commercial or financial relationships that could be construed as a potential conflict of interest.
