# Supplementary material for: A Simple Bayesian Method for Evaluating Whether Data From Patients With Rheumatic Diseases Who Have Been Under Chronic Hydroxychloroquine Medication Since Before the COVID-19 Outbreak Can Speak to Hydroxychloroquine's Prophylactic Effect Against Infection With SARS-CoV-2
Source: Front Med (Lausanne). 2020 Aug 13;7:490. doi: 10.3389/fmed.2020.00490 (PMC7438534; doi:10.3389/fmed.2020.00490)
Supplement: Supplementary file 1 [file Data_Sheet_1.pdf]

Appendix

R script used to derive the results presented in this study (a base version or R is all that is required).

```
#enter population prevalence here (line below)
prevalenceC19 <- 0.01403412

#enter number of HCQpa here (line below)
N_of_HCQpa <- 2000

#enter SARS-CoV-2 test sensitivity here (line below)
sensitivity <- 0.995

#enter SARS-CoV-2 test specificity here (line below)
specificity <- 0.9975


Nmin <- seq(0, N_of_HCQpa )
N_HCQpa <- rep(N_of_HCQpa , N_of_HCQpa +1)
NminusNmin <- N_HCQpa - Nmin
this_less_than0p005 <- 1-pbeta(prevalenceC19, Nmin, NminusNmin)
mode <- (Nmin-1)/(N_HCQpa -2)
theNmin_maxprob <- max(this_less_than0p005[(this_less_than0p005 > 0) & (this_less_than0p005 < 0.005)])
numeralstoHCQpa  <- seq(1,N_of_HCQpa +1)
x <- numeralstoHCQpa [this_less_than0p005==theNmin_maxprob]
if (sensitivity == specificity ){
maxHCQpa  <- Nmin[x]
print("test's sensitivity and specificity are supposed equal, check it is indeed the case!")
print("you entered a sensitivity and specificity value of")
print(sensitivity)
```

```
print("with a COVID-19 population prevalence of")
print(prevalenceC19)
print('and a number of COVID-19 infected HCQpa available of')
print(N_of_HCQpa)
print('the max number of COVID-19 infected HCQpa in order to conclude to a prophylactic effect of HCQ is')
print(maxHCQpa)
print('for that number of COVID-19 infected HCQpa, modal  HCQpa prevalence is (per 100,000)')
print(round(mode[x]*100000,3))
} else {
correction <- round(N_of_HCQpa * (sensitivity - specificity))
x <- x + correction
maxHCQpa  <- Nmin[x]
print("test's sensitivity and specificity are not equal, CORRECTION APPLIED")
print("please check that sensitivity and specificity values are indeed the following:")
print("sensitivity:")
print(sensitivity)
print("specificity:")
print(specificity)
print('with a COVID-19 population prevalence of')
print(prevalenceC19)
print('and a number of COVID-19 infected HCQpa available of')
print(N_of_HCQpa)
print('the max number of COVID-19 infected HCQpa in order to conclude to a prophylactic effect of HCQ is')
print(maxHCQpa)
print('for that number of COVID-19 infected HCQpa, modal  HCQpa prevalence is (per 100,000)')
print(round(mode[x]*100000,3))
}
```
